# Treatment patterns and healthcare resource utilization in palmoplantar pustulosis patients in Japan: A claims database study

**DOI:** 10.1371/journal.pone.0232738

**Published:** 2020-05-22

**Authors:** Celine Miyazaki, Rosarin Sruamsiri, Jӧrg Mahlich, Wonjoo Jung

**Affiliations:** 1 Health Economics Department, Janssen Pharmaceutical K.K., Tokyo, Japan; 2 Department of Pharmacy Practice, Center of Pharmaceutical Outcomes Research, Faculty of Pharmaceutical Sciences, Naresuan University, Phitsanulok, Thailand; 3 Health Economics & Outcomes Research, Janssen-Cilag GmbH, Neuss, Germany; 4 Düsseldorf Institute for Competition Economics (DICE), University of Düsseldorf, Düsseldorf, Germany; University of Toronto, CANADA

## Abstract

**Background:**

Palmoplantar pustulosis (PPP) is a chronic, relapsing, inflammatory autoimmune condition, characterized by sterile pustules on the palms and soles. The treatment patterns of PPP and total health care resource utilization in Japan are not well described. Investigating these areas is needed to understand current PPP management in Japan.

**Objective:**

To describe the characteristics, medication treatment and health care resource utilization patterns, and associated costs of PPP patients in Japan.

**Methods:**

A retrospective analysis of insurance claims data was conducted using the Japan Medical Data Center database. Adult Patients with at least two claims with a PPP diagnosis from January 1, 2011 to March 30, 2017 and six months of follow-up after the first diagnosis were included. Patient characteristics described include age, gender, and comorbid conditions. Treatment patterns assessed include the types of treatment, sequence of treatment, and rates of discontinuation, switching, persistence and use of concomitant medications.

**Results:**

A total of 5,162 adult patients met all inclusion criteria. Mean (SD) patient age was 49.7 (11.6) years and 43.2% were male. A total of 2441 patients (47.8%) received systemic non-biologic drugs during the entire follow up period, 2,366 (46.4%) were prescribed topical therapy, 273 (5.4%) were prescribed phototherapy, while 18 (0.4%) of patients with other autoimmune comorbidities were eligible for prescribed biologics. For treatment-naïve patients with mild PPP, topical therapy was most commonly (77.1%) prescribed, whereas in moderate to severe cases of PPP, systemic non-biologic drugs (65%) were most often used. The frequency of switching was similar (64.3% to 75.3%) across various therapies and treatment lines.

**Conclusion:**

This study describes the treatment patterns and health care resource utilization for Japanese PPP patients using a large claims database, and highlights an unmet need to derive better treatment strategies for PPP and address disease burden in Japan.

## Introduction

Palmoplantar pustulosis (PPP) known also as palmoplantar pustular psoriasis, pustulosis palmoplantaris, or pustulosis palmaris et plantaris; is a chronic, relapsing, autoimmune inflammatory condition, characterized by 1–10 mm intraepidermal sterile pustules on the palms and soles, and presents with frequent, yellow-brown macules (remnants of resolving pustules), erythema, hyperkeratosis, scaling, and fissures [1, 2]. There had been dissension regarding whether PPP represents a distinct skin disorder or a variant of psoriasis [3–6]; however in 2007, PPP was identified as a discrete skin disorder by the International Psoriasis Council due to its localized manifestation [2, 7, 8]. The worldwide prevalence of PPP was estimated to be 0.01–0.05% [2, 9] and in Japan, data from the national health insurance claims database showed a prevalence rate of 0.12%, which is higher than that in the Western population [10].

PPP is closely associated with focal infections, such as tonsillitis, chronic sinusitis and dental infection; in particular, tonsillitis often induces or exacerbates PPP [11]. Other risk factors such as tobacco smoking, autoimmune thyroid disease, trauma, seasonal conditions, certain medications, and psychological stress have been associated with triggering or worsening PPP [3, 7, 12, 13]. Despite the limited area of skin (less than 5%) typically affected, PPP can have significantly greater negative effects on patients’ comfort and quality of life [14] than other forms of inflammatory skin lesions located elsewhere on the body.

Defining optimal therapy for PPP patients has been a constant challenge; standard approaches to therapy, including topical corticosteroids, topical vitamin D3 analogs, oral cyclosporine A, psoralen plus ultraviolet A therapy (PUVA), and narrowband ultraviolet (UV) B [15, 16], have focused mainly on management of symptoms. Usually phototherapy or systemic treatment options like retinoids, methotrexate or cyclosporine A are used for moderate and severe PPP, however, evidence regarding the risks and benefits of these treatments are scarce [17, 18]. In the absence of PPP treatment guidelines to date, PPP patients have not been receiving optimal medical treatments and disease management [19]. Generally, treatment sequence is determined based on severity of signs and symptoms, and underlying risk factors. However, guidance and measures to best characterize severity of PPP are limited, and there is little information on maintenance of remission once achieved [20, 21].

Data on direct medical cost burden for PPP are scarce. Available cost information may be estimated from global costs of psoriasis, which are commensurate with the cost of other critical disorders, such as pancreatic cancer, melanoma, prostate cancer and asthma [22]. Based on systematic literature reviews of cost analyses for psoriasis, the total financial impact of disease is mainly dictated by direct medical costs (e.g. hospitalization, physician’s consultations/visits, medications, over-the-counter prescription, personal care products), phototherapy, and laboratory tests [22, 23]. Thus, it is necessary to evaluate both treatment patterns and medical utilization parameters that drive clinical practice to assess total medical cost burden for PPP.

Currently, there are notable gaps in the literature regarding comorbid health and physical conditions, treatment patterns, persistency and rates of switching to or augmentation with alternative therapies in PPP. To help address these gaps, this retrospective cohort study using an insurance claims database was conducted to describe the characteristics, treatment patterns, health care resource utilization and associated costs in Japanese PPP patients.

## Methodology

### Data source

Data for this retrospective cohort study were retrieved from the Japan Medical Data Center (JMDC) database that comprises retrospective claims data for health insurance reimbursement from medical institutions and pharmacies submitted since January 2005 [24]. Approximately 5.6 million insured persons (approximately 4% of the Japanese population) are included in the database in 2019. The information available in the JMDC database is anonymized, with all personally identifiable information de-identified to protect patient privacy, and the database has been widely used in health services research to investigate various conditions, such as schizophrenia, depression, dermatologic disorders and cardiovascular disease [25, 26]. The retrieved monthly claims information is predominantly from persons of working age (i.e. less than 75 years old) employed by middle- to large-sized companies, as well as their dependents, under the Japanese union-managed health insurance system (Health Insurance Association). Patient medical consultation information can be continuously tracked in chronological order, even if patients attend multiple medical institutions or following hospital transfers, as long as the individual is linked to the Health Insurance Association. The JMDC database provides each patient’s health plan enrollment and demographics information. Records of inpatient and outpatient medical claims (e.g. International Classification of Diseases (ICD)-10 diagnosis codes, procedure codes and cost) and pharmacy claims with prescription dates, drug codes or dosage can be retrieved.

This study was approved by the sponsor’s internal approval committee, Dermatology Research Concept Approval Team and was conducted in accordance with Japanese ethical and legal guidelines. Informed consent was not applicable based on the Ethical Guidelines for Epidemiological Research issued by the Japanese Ministry of Health, Welfare and Labor, given use of de-identified data.

### Patient selection and study design

**Fig 1:**
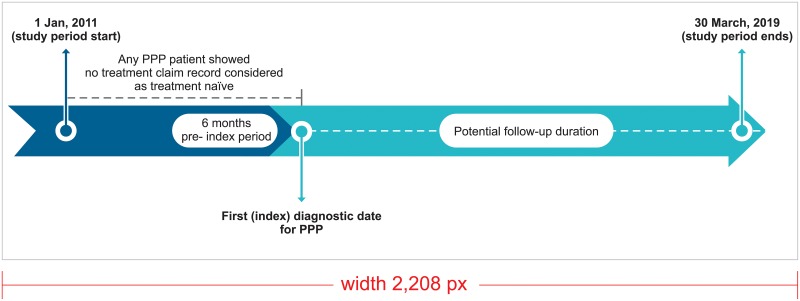
Study scheme.

**Fig 2:**
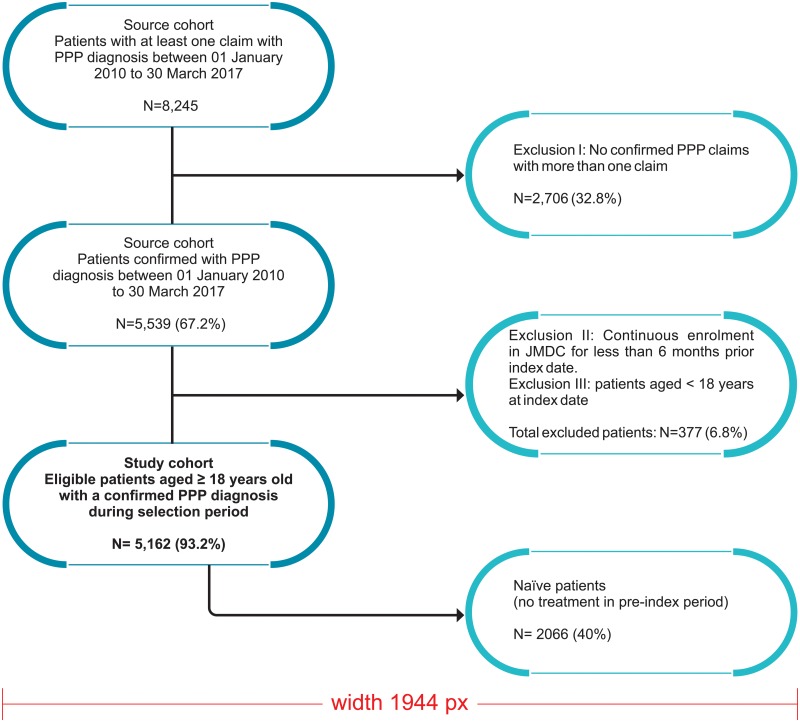
Inclusion criteria and sample attrition. Abbreviations: PPP, Palmoplantar Pustulosis, ICD-10, International Statistical Classification of Diseases and Related Health Problems 10th Revision.

A summary of the overall study design is provided in Fig 1. The study spanned a longitudinal period of 6 years between 01 January, 2011 and 30 March 2017. The index date was defined as “the first claim for a PPP”. To be eligible for selection, a period of at least 6 months of continuous enrollment prior to the index date was required for all pateints. PPP patients with no record of any treatment claim during the continousos enrollment period (i.e. wash-out period) before the index date were considered to be treatment-naïve. A short description of the inclusion and the exclusion criteria is provided in Fig 2.

### Inclusion criteria

Patients were eligible to be included in the study if they were ≥18 years of age, had a confirmed PPP diagnosis i.e. at least two ICD-10 diagnosis codes for PPP or pustulotic arthro-osteitis (PAO) (ICD-10 code: L40.3 are designated for PPP and PAO), had at least 6 months of continuous insurance coverage/enrollment before the index date, and had at least 6 months of continuous insurance coverage/enrollment after the index date to ensure adequate follow-up on study measures.

### Exclusion criteria

Patients were excluded if they had no confirmed diagnosis of PPP, were under 18 years of age at the time of the index date, had continuous enrollment in the database for less than 6 months prior to the index date, or had a specific disease condition that required intensive or special clinical management.

### Study measures

All demographic characteristics available in each patient’s claims data set, including age and sex, were documented. The PPP cohort extracted from the claims database was divided into two groups; one comprising a treatment-naïve cohort (defined based on having had no treatment in the pre-index period) and a treatment-experienced cohort (defined based on having been on any treatment prior to the index date). There are no standardized clinical guidelines established to define or assess severity of PPP in Japan. Subgrouping was based on previously published literature; treatment for patients was used as a proxy for severity [10]. Patients were subdivided into two main severity classes based on assumptions that those who received topical therapy only had mild PPP, while others receiving phototherapy (and/or one or more systematic therapies) had moderate to severe PPP.

### Mild cases

- Patients diagnosed with PPP were classified as having mild disease if they received topical therapy only or did not meet moderate to severe criteria.

### Moderate to severe cases

- Patients diagnosed with PPP were classified as having moderate to severe disease if they received topical therapy, phototherapy, and/or one or more systematic therapies during follow-up using the following treatment categories:
- **Antibiotics (for more than 1 month):** including macrolides and similar types, and tetracycline and derivatives
- **Retinoids:** Etretinate (e.g. systemic therapy used), adapalene, retinol (e.g. high dose of Vitamin A)
- **Topical steroids including systemic steroids (classification by oral or injection administration):** Betamethasone/d-chlorpheniramine maleate, fludrocortisone acetate, betamethasone, dexamethasone, methylprednisolone, prednisolone, triamcinolone, hydrocortisone
- **Non-biologic systemic therapies:** Cyclosporin (CyA), biotin (injection only), diaminodiphenyl sulphone/ diaphenylsulfone, methotrexate, bisphosphonates, sulfasalazine
- **Biologics:** Abatacept, adalimumab, certolizumab pegol, etanercept, golimumab, infliximab, secukinumab, tocilizumab, ustekinumab

### Identification of treatment patterns

Prescriptions for PPP were retrieved using the JMDC Drug tables, in which all drug prescriptions were recorded and identified according to Anatomical Therapeutic Chemical (ATC) codes. The following treatment prescriptions were considered in this study: phototherapy, topical therapy and systemic therapy, including non-biologic and biologic drugs. Records of biologic drug prescriptions were maintained for PPP patients diagnosed with other immunological diseases because no biologic therapy was indicated for PPP until November 2018. Sequential prescriptions recorded during the study (after the first PPP diagnosis identified) were selected and used to define treatment lines. An algorithm was developed to identify treatment lines based on several assumptions in reference to dermatological treatment guidelines [27, 28]. For topical therapy, it was assumed that one prescription of a topical drug would last for 2 weeks (i.e. 14 days) [16]. If the prescription date was missing, an estimated prescription date would be made based on the date when the related claim was recorded. Since, phototherapy is conducted on a regular basis; one exposure was assumed to be effective for 4 weeks [27].

Certain assumptions were made for overlapping treatments in order to define cutoff points for determining whether a patient initiated or switched to a new therapy. It was assumed that the overlap between a topical therapy and other topical drug classes would not be considered a period of concomitant therapy. However, it was assumed that: a) patients would have stopped a topical therapy as a first line treatment when oral therapy or phototherapy was initiated and b) overlap between topical or systemic steroid (e.g. corticosteroids) and other types of treatment could occur because discontinuation of corticosteroid treatment may require progressive dose tapering. Moreover, a period of overlap between phototherapy and systemic drug therapy of longer than 14 days was considered combination therapy.

To identify start dates and end dates for treatment lines for each patient and calculate duration of therapy, Daily Defined Dosage (DDDs) was used as a reference [29]. Once start dates and end dates were defined, patients with duration of treatment not exceeding the follow-up period were categorized as (temporarily) discontinued treatment, switched to another treatment(s), or having an add-on or a reduction in treatment line. A line of treatment was considered ‘discontinued’ if there was no prescription renewal for a period longer than the maximum allowed gap duration (MAGD), also termed the ‘grace period’. A grace period of 61 days was used for all treatment lines [28, 30].

Patients were also characterized based on treatment patterns assessed from the study index date until the earliest date of health plan disenrollment or the end of the JMDC database extraction period (March 30, 2017), as indicated below:

#### Discontinuation

The proportion of patients with discontinuation and time to discontinuation were documented within the specified “grace period’ after the end of the treatment line.

#### Switch

The proportion of patients with a switch, the distribution of alternative agent(s) switched to, and time to switch were assessed.

#### Add on

The proportion of patients with treatment augmentation (combination of two or more drugs), and the distribution of alternative agent(s) added before a grace period of 61 days were assessed.

### Resource utilization

In addition to treatment pattern measures, specific health-care resource utilization (HCRU) measures were assessed for each patient [31]. These measures included outpatient/physician office visits, hospitalizations, and pharmacy claims. The proportion of patients with at least one occurrence was calculated and reported by type of resource utilization during the overall follow-up period. First occurrence for the healthcare resource category of interest was the target event, and censoring occurred if a patient reached the end of the follow-up period without any claim related to this type of resource. Proportions of patients remaining on treatment or using medical resources before discontinuing or without event occurrence at last follow-up (e.g. censored data) were estimated using Kaplan-Meier methodology. The average number of occurrences per person per month was calculated and reported by type of resource utilization. For each measure of HCRU, we divided the sum of the number of occurrences for each patient by the sum of their follow-up time (in month). Patients contributed to HCRU rates only while they were alive and were included in the health care database.

### Direct medical cost evaluation

Cost data were extracted from the JMDC database and were reported in Japanese yen (¥). PPP-related resource utilization and costs included encounters and payments associated with claims containing a diagnosis code for PPP (ICD-10 codes L40.3) or pharmacy claims for PPP-related medications. For each resource-used category, the sum of the costs for patients was divided by the sum of the patient’s follow-up time (in month). Patients contributed to these rates only when they were alive and in the health plan.

### Statistical analysis

Baseline demographics and characteristics of Japanese PPP patients were reported descriptively. Proportions and numbers of PPP patients, and data for each group and parameter were calculated by descriptive statistical methods. Summary statistical values were expressed in mean and standard deviation (SD) or median with inter quartile range for continuous variables and frequency distribution for categorical variables. Kaplan-Meier curves were used to evaluate time to discontinuation of medical resource used. All statistical analyses were conducted by using SAS 9.3 software (SAS institute Inc., Cary, NC, USA).

## Results

### Patient characteristics

There were 8,245 patients who had at least one claim for a PPP diagnosis From January 1, 2010 to March 30, 2017 (Fig 2). Among these, 5,539 patients had a diagnosis of PPP confirmed with two claims. The demographic characteristics of PPP patients are summarized in Table 1. Of a total of 5,162 eligible patients with at least 6 months of continuous health plan enrollment before and post-index date, 2,066 (40%) were treatment-naïve, with no treatment within the six months prior to the index date. In turn, 3,042 (58.9%) were treatment-experienced, and 54 (0.1%) had no prescription for PPP treatment during the study period. Of the total number of patients included, 43.2% were male and mean (SD) age was 49.7 (11.6) years. The prevalence of PPP was highest (30.4%) among middle-aged individuals between 45 to 54 years.

**Table 1:** Patient demographics and co-morbidities in the 6-month pre-index period and during selection period.

Baseline characteristics	All patients			Naïve patients		
	Total	Mild	Moderate to severe	Total	Mild	Moderate to severe
	N = 5162	N = 1782	N = 3380	N = 2066	N = 838	N = 1228
Follow-up duration (days), Mean (SD)	1064.7 (690.5)	891.1 (654.5)	1156.2 (691.5)	992.9 (655.1)	877.4 (635.8)	1071.7 (656.5)
Age at index date, Mean (SD)	49.7 (11.6)	50.1 (12.1)	49.5 (11.4)	49.3 (11.7)	49.6 (12.1)	49.1 (11.4)
Age at index date by categories, n (%)						
18–34	597 (11.6)	209 (11.7)	388 (11.5)	251 (12.1)	106 (12.6)	145 (11.8)
35–44	1046 (20.3)	353 (19.8)	693 (20.5)	420 (20.3)	164 (19.6)	256 (20.8)
45–54	1568 (30.4)	504 (28.3)	1064 (31.5)	644 (31.2)	248 (29.6)	396 (32.2)
55–64	1508 (29.2)	538 (30.2)	970 (28.7)	587 (28.4)	244 (29.1)	343 (27.9)
65+	443 (8.6)	178 (10.0)	265 (7.8)	164 (7.9)	76 (9.1)	88 (7.2)
Gender: Male, n (%)	2229 (43.2)	858 (48.1)	1371 (40.6)	890 (43.1)	108 (38.7)	782 (43.8)
Multiple medications (ATC code level 3) ^a^						
Mean (SD)	6.3 (6.1)	5.2 (5.3)	6.9 (6.4)	2.6 (3.8)	2.8 (3.8)	2.5 (3.8)
Median	5.0	4.0	6.0	1.0	1.0	0.0
Multiple medications (ATC code level 3) by categories of number of drugs, n (%)						
0	1047 (20.3)	393 (22.1)	654 (19.3)	991 (48.0)	358 (42.7)	633 (51.5)
[1–3]	978 (18.9)	445 (25.0)	533 (15.8)	482 (23.3)	221 (26.4)	261 (21.3)
[4–5]	751 (14.5)	266 (14.9)	485 (14.3)	242 (11.7)	101 (12.1)	141 (11.5)
[6–8]	624 (12.1)	205 (11.5)	419 (12.4)	132 (6.4)	59 (7.0)	73 (5.9)
> 8	1762 (34.1)	473 (26.5)	1289 (38.1)	219 (10.6)	99 (11.8)	120 (9.8)
Multiple medications (ATC code level 1)						
Mean (SD)	3.8 (3.0)	3.3 (2.7)	4.1 (3.1)	1.8 (2.2)	1.9 (2.2)	1.7 (2.2)
Median	4.0	3.0	4.0	1	1.0	1.0
Multiple medications (ATC code level 1) by categories of number of drugs, n (%)						
0	966 (18.7)	363 (20.4)	603 (17.8)	946 (45.8)	344 (41.1)	602 (49.0)
[1–3]	1580 (30.6)	671 (37.7)	909 (26.9)	700 (33.9)	319 (38.1)	381 (31.0)
[4–5]	1132 (21.9)	363 (20.4)	769 (22.8)	258 (12.5)	99 (11.8)	159 (12.9)
[6–8]	817 (15.8)	239 (13.4)	578 (17.1)	116 (5.6)	54 (6.4)	62 (5.0)
> 8	667 (12.9)	146 (8.2)	521 (15.4)	46 (2.2)	22 (2.6)	24 (2.0)
CCI						
Mean (SD)	0.4 (0.9)	0.3 (0.8)	0.4 (0.9)	0.2 (0.7)	0.3 (0.7)	0.2 (0.7)
Median	0.0	0.0	0.0	0.0	0.0	0.0
Comorbidity at baseline, n (%)						
Rheumatoid arthritis	104 (2.0)	10 (0.6)	94 (2.8)	14 (0.7)	4 (0.5)	10 (0.8)
Inflammatory bowel disease	76 (1.5)	16 (0.9)	60 (1.8)	15 (0.7)	6 (0.7)	9 (0.7)
Ankylosing spondylitis	3 (0.1)	0 (0.0)	3 (0.1)	0 (0.0)	0 (0.0)	0 (0.0)
Juvenile arthritis	0 (0.0)	0 (0.0)	0 (0.0)	0 (0.0)	0 (0.0)	0 (0.0)
Psoriasis	164 (3.2)	36 (2.0)	128 (3.8)	0 (0.0)	0 (0.0)	0 (0.0)
Comorbidity at selection period n (%)						
Rheumatoid arthritis during selection period	248 (4.8)	22 (1.2)	226 (6.7)	64 (3.1)	8 (1.0)	56 (4.6)
Inflammatory bowel disease during selection period	195 (3.8)	33 (1.9)	162 (4.8)	50 (2.4)	13 (1.6)	37 (3.0)
Ankylosing spondylitis during selection period	7 (0.1)	1 (0.1)	6 (0.2)	1 (0.0)	0 (0.0)	1 (0.1)
Juvenile arthritis during selection period	1 (0.0)	0 (0.0)	1 (0.0)	1 (0.0)	0 (0.0)	1 (0.1)
Psoriasis during selection period	602 (11.7)	155 (8.7)	447 (13.2)	183 (8.9)	74 (8.8)	109 (8.9)
Treatment experience						
naïve	2066 (40.0)	838 (47.0)	1228 (36.3)	2066 (100.0)	838 (100.0)	1228 (100.0)
Experienced	3042 (58.9)	894 (50.2)	2148 (63.6)	0 (0.0)	0 (0.0)	0 (0.0)
NA	54 (1.0)	50 (2.8)	4 (0.1)	0 (0.0)	0 (0.0)	0 (0.0)
Medical intervention experience						
GMA: Granulocyte Monocyte Apheresis	1 (0.0)	1 (0.1)	0 (0.0)	0 (0.0)	0 (0.0)	0 (0.0)
Tonsillectomy	32 (0.6)	7 (0.4)	25 (0.7)	12 (0.6%)	5 (0.6)	7 (0.6)

^a^: Multiple medication were evaluated by the number of drugs prescribed (by 3-digit ATC classes).

CCI: Charlson Comorbidity Index.

NA: Not applicable.

Among patients in this PPP cohort, 34.5% were categorized as having mild PPP, while 65.4% were categorized as having moderate to severe disease. With regard to medications other than PPP-approved drugs, the mean (SD) numbers of ATC Level 3 and Level 1 drugs prescribed were 6.3 (6.1) and 3.8 (3.0), respectively. Of the total number of PPP patients included, 1762 (34.1%) received > 8 different drugs classified as ATC Level 3, which largely reflected the group of patients with moderate to severe disease (1289 38.1). Moreover, 1580 (30.6%) patients received 1–3 different drugs classified as ATC Level 1, which similarly reflected both the mild and moderate to severe groups of PPP patients. Using the Charlson comorbidity index (CCI) as a measure, the mean (SD) comorbidity burden was 0.4 (0.9). In addition, 11.7% of PPP patients also had psoriasis, 4.8% had rheumatoid arthritis, 3.8% had inflammatory bowel disease, and 0.1% had ankylosing spondylitis. Across the overall PPP population, 32 (0.6%) of patients had a tonsillectomy, and one patient received monocyte adsorptive apheresis (GMA) treatment.

### Treatment patterns

Japanese PPP patients were prescribed various types of treatment during the post-index period, as outlined in Table 2. Among all 5,162 included patients, 2,366 (46.4%) were prescribed topical therapy, 273 (5.4%) received phototherapy, 2,441 (47.8%) were prescribed non-biologic systematic drugs, and 18 (0.4%) received biologic therapy during the follow up period. Among treatment-naive patients, 1,228 (59.4%) had moderate to severe disease, and 780 (63.5%) received a non-biologic systemic therapy as their first line treatment after their PPP diagnosis was established. Two treatment-naive PPP patients received biologic therapy as first line treatment; however, these patients received biologic therapy primarily for another autoimmune condition, as biologics were not approved for PPP within the time frame of the study.

**Table 2:** Treatments patterns in patients based on severity level of PPP.

Characteristics	All patients			Naïve patients		
	Total	Mild	Moderate to severe	Total	Mild	Moderate to severe
	N = 5162	N = 1782	N = 3380	N = 2066	N = 838	N = 1228
Patients with least 1 PX^a^	5108 (99.0)	1732 (97.2)	3376 (99.9)	2066 (100.0)	838 (100.0)	1228 (100.0)
First line						
No treatment	0	0	0	0	0	0
Topical therapy	2366 (46.4)	1331 (77.1)	1035 (30.7)	1056 (51.2)	647 (77.6)	409 (33.3)
Phototherapy	273 (5.4)	147 (8.5)	126 (3.7)	101 (4.9)	64 (7.6)	37 (3.0)
Non-biologic systemic drugs	2441 (47.8)	246 (14.2)	2195 (65.0)	902 (43.7)	122 (14.6)	780 (63.5)
Biologic drugs ^b^	18 (0.4)	0	18 (0.5)	2 (0.1)	0	2 (0.2)
GMA	0	0	0	0	0	0
Tonsillectomy	4 (0.1)	3 (0.2)	1 (0.0)	2 (0.1)	2 (0.2)	0

^a^ Prognosis,

^b^ Biologics were not use for PPP condition at this investigation time frame,

^C^ Three naïve patients’ treatment record were missing at first line in the claims database. Total number of patients in the cohort: N; data are reported as N (%) percentages.

A detailed description of mutually exclusive treatments and combination therapies employed for PPP, constituting six treatment lines, is shown in Table 3. The various treatment lines used were examined for treatment-naïve patients only. Among all treatment naïve patients, phototherapy appeared to be more commonly used when prescribed in combination with topical corticosteroids. Overall, 51.3% of the treatment-naïve patients received topical therapy and 43% received non-biologic systemic drugs as first treatment line. Similar proportions were observed across all treatment lines. Non-biologic systemic drugs were also commonly used in combination with topical therapies across all lines of therapy, however the proportion of PPP patients receiving non-biologic systemic drugs in combination with topical therapy was slightly lower compared to the proportion of patients receiving non-biologic systemic drug only. Patterns of prescription of various therapies based on severity of PPP are provided in S1 Table. A supplementary list of drugs prescribed in treatment-naive PPP patients through up to the 6^th^ line of treatment is shown in S2 Table.

**Table 3:** Overall treatment patterns of treatment-naïve patients from the first line treatment to the sixth line treatment PPP.

Treatment line	1st line	2nd-line	3rd line	4th line	5th line	6th line
All treatment naïve patients	N = 2066	N = 1458	N = 1010	N = 702	N = 451	N = 296
***Topical therapy***	**1059 (51.3)**	**722 (49.5)**	**491 (48.6)**	**353 (50.3)**	**217 (48.1)**	**141 (47.6)**
***Phototherapy***	**101 (4.9)**	**73 (5.0)**	**43 (4.3)**	**24 (3.4)**	**12 (2.7)**	**12 (4.1)**
Without any additional prescription	9 (8.9)	12 (16.4)	4 (9.3)	6 (25.0)	3 (25.0)	4 (33.3)
With Topical therapy	92 (91.1)	61 (83.6)	39 (90.7)	18 (75.0)	9 (75.0)	8 (66.7)
***Non-biologic systemic drugs***	**902 (43.7)**	**657 (45.1)**	**470 (46.5)**	**325 (46.3)**	**221 (49.0)**	**142 (48.0)**
Without any additional prescription	151 (16.7)	330 (50.2)	279 (59.4)	194 (59.7)	139 (62.9)	93 (65.5)
With Topical therapy	624 (69.2)	285 (43.4)	169 (36.0)	121 (37.2)	73 (33.0)	42 (29.6)
With phototherapy	2 (0.2)	2 (0.3)	3 (0.6)	2 (0.6)	1 (0.5)	1 (0.7)
With Topical therapy and phototherapy	125 (13.9)	40 (6.1)	19 (4.0)	8 (2.5)	8 (3.6)	6 (4.2)
***Biologic drugs***	**2 (0.1)**	**2 (0.1)**	**3 (0.3)**	**0 (0.0)**	**0 (0.0)**	**1 (0.3)**
Without any additional prescription	0 (0.0)	1 (50.0)	0 (0.0)	0 (0.0)	0 (0.0)	0 (0.0)
With non-biologic drugs	1 (50.0)	1 (50.0)	1 (33.3)	0 (0.0)	0 (0.0)	1 (100.0)
With phototherapy	0 (0.0)	0 (0.0)	0 (0.0)	0 (0.0)	0 (0.0)	0 (0.0)
With Topical therapy	0 (0.0)	0 (0.0)	1 (33.3)	0 (0.0)	0 (0.0)	0 (0.0)
With Topical therapy and phototherapy	0 (0.0)	0 (0.0)	0 (0.0)	0 (0.0)	0 (0.0)	0 (0.0)
With non-biologic drugs and phototherapy	1 (50.0)	0 (0.0)	1 (33.3)	0 (0.0)	0 (0.0)	0 (0.0)
With prescriptions of topical and non-biologics	0 (0.0)	0 (0.0)	0 (0.0)	0 (0.0)	0 (0.0)	0 (0.0)
With prescriptions of topical, non-biologics drugs and Phototherapy	0 (0.0)	0 (0.0)	0 (0.0)	0 (0.0)	0 (0.0)	0 (0.0)
***Medical intervention***						
GMA	0 (0.0)	0 (0.0)	0 (0.0)	0 (0.0)	0 (0.0)	0 (0.0)
Tonsillectomy	2 (0.1)	4 (0.3)	3 (0.3)	0 (0.0)	1 (0.2)	0 (0.0)

### Treatment-related events during therapy

The most frequent change in treatment pattern among treatment-naïve patients was switching to a different regimen (Table 4). Non-biologic systemic drugs had the longest duration of therapy (mean, 178.51 days [SD 309.1]) when used as first line treatment, followed by phototherapy (mean, 141.58 days [SD 157.9]), biologics (mean, 135.5 [SD 47.4]) and topical therapy (mean, 96.2 days [SD 138.4]). Across use as 1st to 3rd lines of treatment, switching from non-biologic systemic drugs occurred slightly less frequently compared to switching from topical and phototherapy. Regardless of treatment line or severity of PPP, switching rather than discontinuation was the most common treatment-related event (S3 Table). No instances of add-on or reduction of therapies were documented across all treatment lines.

**Table 4:** Duration of treatment lines, number of treatment-related events, according to the treatment regimen dispensed.

	Topical therapy	Phototherapy	Non-biologic systemic drugs	Biologic drugs^a^
**1st treatment line**	**N = 1056** ^b^	**N = 101**	**N = 902**	**N = 2**
Duration of the line (days)				
Mean (SD)	96.2 (138.4)	141.58 (157.9)	178.51 (309.1)	135.5 (47.4)
Median	53	98	69.5	135.5
Treatment-related events				
> Discontinuation	189 (17.9%)	11 (10.9%)	147 (16.3%)	0
> Switch	793 (75.1%)	76 (75.3%)	587 (65.1%)	1 (50.0%)
> Add-on	0	0	0	0
> Reduction	0	0	0	0
> No change	74 (7.0%)	14 (13.9%)	168 (18.6%)	1 (50.0%)
**2nd treatment line**	**N = 722**	**N = 73**	**N = 657**	**N = 2**
Duration of the line (days)				
Mean (SD)	80.1 (126.8)	130.3 (178.6)	85.3 (231.0)	173.0 (202.2)
Median	28	76	14	173
Treatment-related events				
> Discontinuation	143 (19.8%)	9 (12.3%)	134 (20.4%)	1 (50.0%)
> Switch	501 (69.4%)	52 (71.2%)	453 (69.0%)	1 (50.0%)
> Add-on	0	0	0	0
> Reduction	0	0	0	0
> No change	78 (10.8%)	12 (16.4%)	70 (10.7%)	0
**3rd treatment line**	**N = 491**	**N = 43**	**N = 470**	**N = 3**
Duration of the line (days)				
Mean (SD)	62.22 (74.3)	108.40 (158.1)	72.69 (189.3)	146.00 (82.2)
Median	28	41	9	162
Treatment-related events				
> Discontinuation	88 (17.9%)	5 (11.6%)	103 (21.9%)	0
> Switch	366 (74.5%)	32 (74.4%)	302 (64.3%)	2 (66.7%)
> Add-on	0	0	0	0
> Reduction	0	0	0	0
> No change	37 (7.54%)	6 (14.0%)	65 (13.8%)	1 (33.3%)

All medical interventions were excluded.

^a^ Biologics were not use for PPP condition at this investigation time frame,

^b^ Three naïve patients’ treatment record were missing at first line in the claims database.

Total number of treatment-naïve patients in the cohort (N); data are reported as N (%) percentages.

### Health care resource utilization and associated costs

**Fig 3:**
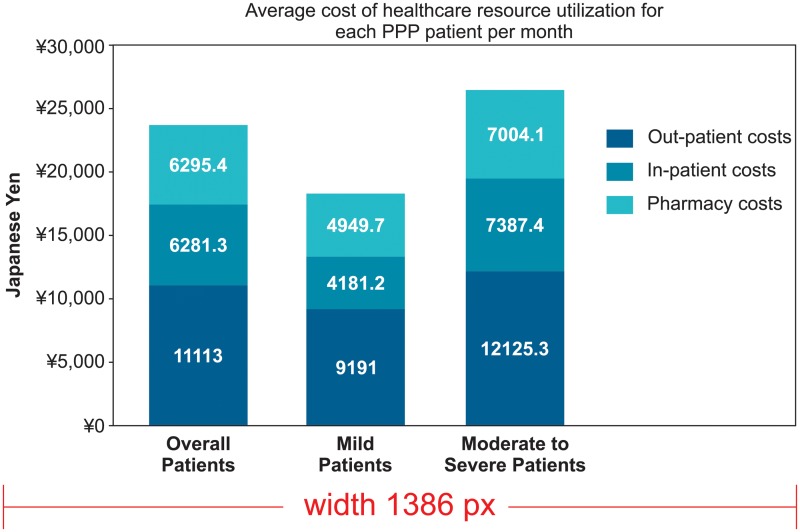
Total health care costs of PPP in Japan estimated for the overall PPP patient cohorts during all follow up period of the study.

Hospitalization and other health care resource utilization patterns recorded are presented in Table 5 and Fig 3. Most of the patients with PPP (99.6%), inclusive of all mild and moderate to severe cases of PPP, had at least one outpatient visit, with a mean (SD) number of visits per patient of 3.08 (3.06) per month during the follow-up period. Mean (SD) outpatient costs per patient per month in the post-index period were ¥ 11,113.0 (¥21364.1). Nearly 14.4% of patients had at least one PPP-related hospitalization during the period of study. Among those patients who were hospitalized, the mean (SD) number of admissions per patient per month was 0.05 (0.08), with a mean (SD) inpatient cost per patient per month of ¥6,281.3 (¥42,255.9). Among the total PPP patients in this analysis, 5.3% had at least one X-ray, 19.8% had at least one MRI, 34.4% had at least one CT scan, and 1.4% had at least one bone scintigraphy study. The mean (SD) pharmacy cost per patient per month in the post-index period was ¥6,295.4 (¥10,209.2). The mean (SD) total/overall cost per patient per month, inclusive of PPP-related hospitalizations, outpatient visits, and pharmacy costs, was ¥23,689.8 (¥54,445.6).

**Table 5:** Patterns of healthcare resource utilization during all follow-up period of the study.

	Overall patients	Mild	Moderate to severe
**Hospitalization**	**N = 5162**	**N = 1782**	**N = 3380**
At least one follow-up visit, N (%)	742 (14.4%)	179 (10.0)	563 (16.7)
No. admission per patient/month*: Mean (SD)	0.05 (0.08)	0.06 (0.08)	0.05 (0.08)
**Outpatient visit**	**N = 5162**	**N = 1782**	**N = 3380**
At least one follow-up visit, N (%)	5143 (99.6)	1771 (99.4)	3372 (99.8)
No. visits per patient/month*: Mean (SD)	3.08 (3.26)	2.59 (2.82)	3.34 (3.44)
**Imaging procedure**	**N = 5162**	**N = 1782**	**N = 3380**
***X-ray***			
At least one occurrence, N (%)	274 (5.3)	66 (3.7)	208 (6.1)
No. occurrences per patient/month*	0.10 (0.14)	0.10 (0.06)	0.10 (0.16)
***MRI***			
At least one occurrence, N (%)	1023 (19.8)	209 (11.7)	814 (24.0)
No. occurrences per patient/month*	0.06 (0.09)	0.06 (0.06)	0.07 (0.09)
***CT scan***			
At least one occurrence, N (%)	1777 (34.4)	452 (25.3)	1325 (39.2)
No. occurrences per patient/month*	0.16 (0.26)	0.17 (0.29)	0.15 (0.25)
***Bone scintigraphy***			
At least one occurrence, N (%)	76 (1.4)	12 (0.6)	64 (1.8)
No. occurrences per patient/month*	0.05 (0.07)	0.09 (0.14)	0.04 (0.04)

*Among patients with at least one occurrence.

## Discussion

Evidence has shown that there remain unmet needs for optimizing treatment of PPP, and there is a necessity for further studies to inform health care providers and patients about the relative merits of the many available treatment options. PPP is particularly prevalent in Japan compared to western countries, and it is also an intractable disease that is often difficult to diagnosis [2, 32]. Thus, it is imperative to understand the current patterns of treatment and healthcare resource utilization for PPP patients, identify management gaps, and address the economic burden of this autoimmune disorder. To our knowledge, no other study to date has assessed health care resource utilization and cost burden for patients with PPP.

This study determined that topical therapy and non-biologic systemic therapy are the two most commonly used forms of treatment across all lines of treatment options for PPP. Moreover, our study showed that phototherapy was most commonly used when prescribed in combination with topical corticosteroids, which is similar to findings by Raposo and colleagues [7]. In addition to this, a large proportion of PPP patients, including those who had biologic claims, continued to use topical therapy in combination with non-biologic systematic drugs and phototherapy as well, even after they had initiated a new category of drug treatment. Taken together, these findings imply that PPP patients are prone to relapses even when potent topical steroids are used over an extended period or in combination with other treatments. Moreover, reduction of topical steroid use, when combined with other therapies, was not observed among PPP patients in this cohort. Similarly, another study found that more than half of the PPP patients who received potent topical steroids relapsed after 2 to 16 weeks of continuous treatment [18].

These observations raise the uncertainty on the effectiveness of topical steroids and phototherapy combination for treating moderate to severe PPP [28, 33]. There is currently insufficient evidence on prolonged use of topical therapy in combination with phototherapy, or with non-biologic systemic therapy, for patients with PPP, and thus future studies regarding efficacy and safety of combination therapies are required. While effectively treating PPP continues to be a challenge, no studies have shown that PPP patients who do not benefit from combinations of phototherapy, PUVA and systematic therapy, may be appropriate candidates for biologic therapy [3, 7]. As understanding of the pathophysiology of PPP increases, new therapeutic agents and novel combinations of existing therapies are likely to become options for moderate to severe patients who suffer from intractable disease. The efficacy and safety of biologics used in combination with other systemic therapies is yet to be studied and established [19]; however, this approach may be an option for patients with more extensive palmoplantar disease resistant to standard treatments.

Adherence to and persistence with medications are required for achieving and maintaining improvement of moderate to severe dermatological autoimmune conditions (e.g. psoriasis or PPP) [3, 34]. The findings from our study indicate that switching within drug class was the most common action taken among patients, irrespective of the severity of PPP in treatment-naïve patients. Non-biologic systemic drugs used for treatment of moderate to severe PPP had the worst persistence, indicating that there is an unmet medical need for more effective therapies. High proportions of treatment-naïve patients receiving topical and non-biologic systemic therapies switched treatment across all lines, which likely reflects limited alternative options with later line therapy. Furthermore, PPP patients who repeatedly switch treatments over time are likely to experience lower quality of life (i.e. clearance of pustules, erythema, hyperkeratosis, scaling, and fissures of the palms and soles) due to lack of achieving significant levels of clinical response [12]. In addition, there are no specific guidelines for treatment of PPP as a separate entity, and guidelines for other conditions, such as generalized pustular psoriasis, are sometimes applied for PPP [35]. Consequently, use of conventional therapies may not be optimized for reducing the clinical burden associated with the disease.

This retrospective claims database study was conducted to assess treatment patterns, health care resource utilization and associated costs for PPP, which may be further impacted by the presence of associated comorbid conditions. Results suggest that 4.8% of patients had rheumatoid arthritis, 3.8% had inflammatory bowel disease and 11.7% had psoriasis as a comorbid health disorder in the 6-month pre-index baseline period. A study in Japanese patients observed that PAO is commonly seen in conjunction with PPP; other common comorbidities associated with PPP include metabolic disorders, hypertension, depression and autoimmune diseases, all of which may also contribute to the impact of PPP on patients’ quality of life [36–38]. Accounting for comorbidity may influence treatment choice, which may be further complicated for patients who have switched multiple therapies in the past.

We also examined the cost of PPP management using this claims database. Our results revealed that total direct medical (i.e. in-patient, out-patient and pharmacy) costs of PPP per month are mostly driven by the number of PPP-related outpatient visits, which are slightly (1.5%) higher for moderate to severe compared to mild PPP patients. More effective therapies may have the potential to limit long-term overall costs by reducing various aspects of patient care, including outpatient visits [39–41]. Among factors that significantly influence the direct costs associated with PPP are lack of standardized treatment regimens, increased frequency of drug switching, unadvised treatment discontinuation, and treatment failures, all of which could have long-term impact.

While new classes of treatment are beginning to emerge for various dermatological autoimmune diseases (e.g. psoriasis or psoriatic arthritis) based on increasing knowledge of pathogenic mechanisms (e.g. the specific immune regulatory pathways or genetic predisposition), it is important to consider that new classes of medications or therapies could potentially benefit PPP patients as well [42]. In Japan, the first biologic therapy for PPP was recently approved, and could represent an important advance in treating patients with PPP. In light of the need to address the unmet needs of patients with intractable dermatological autoimmune diseases such as PPP, evaluating the impact of newer treatments, such as biologics, especially utilizing real-world data to optimize patient management, is warranted.

### Limitations

We used the JMDC database, which, unlike other types of claims databases that are restricted to hospital data, allowed us to track patients longitudinally across different medical institutions. However, the findings of this study should be considered within the context of several limitations inherent to the JMDC data source. The JMDC covers just 4% of the total Japanese population, and therefore it might not constitute a representative sample. Patients may also be receiving health care benefits under other insurance types not captured in this study. In addition, the database captures Japanese PPP patients only up to 75 years of age which a sizable proportion of elderly PPP patients over 75 years of age may have been overlooked.

Other limitations relate to data collection and extraction of information. Loss of productivity and other indirect costs could not be evaluated using information from this claims database. The database does not account for reasons underlying specific treatment-related decisions, such as treatment discontinuation, switching, and augmentation. Classification of severity was made through treatment proxy and based on the approach of Kubota et. al 2015 [10]. Very few biologics were prescribed for PPP patients, and only in the context of treatment for a diagnosis of another immunological diseases. Treatment-related decisions were evaluated from the perspective of the naïve patient cohort only, and it was not possible to capture treatment-related events for treatment-experienced patients before baseline selection. Lastly, our study was descriptive and not comparative in nature. Consequently, interpretation of findings regarding treatment patterns and treatment-related decisions should be considered in the context of these limitations.

## Conclusions

This is the first studies conducted in Japan to evaluate a large cohort exclusively of patients with PPP from an administrative claims database and to assess treatment patterns and health care resource utilization associated with PPP. Treatment persistence and adherence in patients with PPP was found to be poor, which suggests that there is a need for more effective treatment options for PPP. Strategies aimed at improving both medication continuity and adherence have the potential to improve PPP management and burden. As there are no clinical guidelines established in Japan, there may be a need for increased disease management and awareness among Japanese patients and health care providers, which could also reduce unnecessary medical resource unitization and costs. In summary, this study analyzed the current treatment patterns, treatment-related decision making, and economic burden related to PPP in Japan, and supports the need to revisit the management gaps and guideline recommendations for PPP.

## Supporting information

S1 Table Overall treatment patterns based on severity level of PPP (include only treatment naïve patients at index date). (DOCX)

S2 Table List of drugs prescribed to treatment naïve PPP patients up to 6^th^ line of treatment. (DOCX)

S3 Table List of drugs approved for the treatment of PPP in Japan. (DOCX)

S4 Table Overall duration of treatment lines, number of treatment-related events up to 6^th^ line of treatment according to the treatment regimen dispensed, (N) total number of patients in the cohort; data are reported as N (%) percentages. (DOCX)
