# Supplementary material for: Treatment patterns and healthcare resource utilization in palmoplantar pustulosis patients in Japan: A claims database study
Source: PLoS One. 2020 May 22;15(5):e0232738. doi: 10.1371/journal.pone.0232738 (PMC7244105; doi:10.1371/journal.pone.0232738)
Supplement: S1 Table — (DOCX) [file pone.0232738.s001.docx]

**Table S1:** **Overall treatment patterns based on severity level of PPP (include only treatment naïve patients at index date)**

| **Treatment line** | **1st line** | **2nd-line** | **3rd line** | **4th line** | **5th line** | **6th line** |
| --- | --- | --- | --- | --- | --- | --- |
| **Treatment regimen** |  |  |  |  |  |  |
| **All naïve patients** | **N=2066** | **N=1458** | **N=1010** | **N=702** | **N=451** | **N=296** |
| ***Topical therapy*** | **1059 (51.3)** | **722 (49.5)** | **491 (48.6)** | **353 (50.3)** | **217 (48.1)** | **141 (47.6)** |
| ***Phototherapy*** | **101 (4.9)** | **73 (5.0)** | **43 (4.3)** | **24 (3.4)** | **12 (2.7)** | **12 (4.1)** |
| Without any additional prescription | 9 (8.9) | 12 (16.4) | 4 (9.3) | 6 (25.0) | 3 (25.0) | 4 (33.3) |
| With Topical therapy | 92 (91.1) | 61 (83.6) | 39 (90.7) | 18 (75.0) | 9 (75.0) | 8 (66.7) |
| ***Non-biologic drugs*** | **902 (43.7)** | **657 (45.1)** | **470 (46.5)** | **325 (46.3)** | **221 (49.0)** | **142 (48.0)** |
| Without any additional prescription | 151 (16.7) | 330 (50.2) | 279 (59.4) | 194 (59.7) | 139 (62.9) | 93 (65.5) |
| With Topical therapy | 624 (69.2) | 285 (43.4) | 169 (36.0) | 121 (37.2) | 73 (33.0) | 42 (29.6) |
| With phototherapy | 2 (0.2) | 2 (0.3) | 3 (0.6) | 2 (0.6) | 1 (0.5) | 1 (0.7) |
| With Topical therapy and phototherapy | 125 (13.9) | 40 (6.1) | 19 (4.0) | 8 (2.5) | 8 (3.6) | 6 (4.2) |
| ***Biologic drugs*** | **2 (0.1)** | **2 (0.1)** | **3 (0.3)** | **0 (0.0)** | **0 (0.0)** | **1 (0.3)** |
| Without any additional prescription | 0 (0.0) | 1 (50.0) | 0 (0.0) | 0 (0.0) | 0 (0.0) | 0 (0.0) |
| With non-biologic drugs | 1 (50.0) | 1 (50.0) | 1 (33.3) | 0 (0.0) | 0 (0.0) | 1 (100.0) |
| With phototherapy | 0 (0.0) | 0 (0.0) | 0 (0.0) | 0 (0.0) | 0 (0.0) | 0 (0.0) |
| With Topical therapy | 0 (0.0) | 0 (0.0) | 1 (33.3) | 0 (0.0) | 0 (0.0) | 0 (0.0) |
| With Topical therapy and phototherapy | 0 (0.0) | 0 (0.0) | 0 (0.0) | 0 (0.0) | 0 (0.0) | 0 (0.0) |
| With non-biologic drugs and phototherapy | 1 (50.0) | 0 (0.0) | 1 (33.3) | 0 (0.0) | 0 (0.0) | 0 (0.0) |
| With prescriptions of topical and non-biologics | 0 (0.0) | 0 (0.0) | 0 (0.0) | 0 (0.0) | 0 (0.0) | 0 (0.0) |
| With prescriptions of topical, non-biologics drugs and Phototherapy | 0 (0.0) | 0 (0.0) | 0 (0.0) | 0 (0.0) | 0 (0.0) | 0 (0.0) |
| ***Medical intervention*** |  |  |  |  |  |  |
| GMA | 0 (0.0) | 0 (0.0) | 0 (0.0) | 0 (0.0) | 0 (0.0) | 0 (0.0) |
| Tonsillectomy | 2 (0.1) | 4 (0.3) | 3 (0.3) | 0 (0.0) | 1 (0.2) | 0 (0.0) |
| **Severity: Mild** | **N=838** | **N=509** | **N=288** | **N=171** | **N=88** | **N=50** |
| ***Topical therapy*** | **650 (77.6)** | **351 (69.0)** | **210 (72.9)** | **125 (73.1)** | **67 (76.1)** | **39 (78.0)** |
| ***Phototherapy*** | **64 (7.6)** | **30 (5.9)** | **13 (4.5)** | **6 (3.5)** | **4 (4.5)** | **2 (4.0)** |
| Without any additional prescription | 6 (9.4) | 2 (6.7) | 1 (7.7) | 1 (16.7) | 0 (0.0) | 0 (0.0) |
| With Topical therapy | 58 (90.6) | 28 (93.3) | 12 (92.3) | 5 (83.3) | 4 (100.0) | 2 (100.0) |
| ***Non-biologic drugs*** | **122 (14.6)** | **126 (24.8)** | **64 (22.2)** | **40 (23.4)** | **17 (19.3)** | **9 (18.0)** |
| Without any additional prescription | 46 (37.7) | 94 (74.6) | 55 (85.9) | 38 (95.0) | 15 (88.2) | 8 (88.9) |
| With phototherapy | 1 (0.8) | 0 (0.0) | 0 (0.0) | 0 (0.0) | 0 (0.0) | 0 (0.0) |
| With Topical therapy | 65 (53.3) | 30 (23.8) | 7 (10.9) | 2 (5.0) | 2 (11.8) | 1 (11.1) |
| With Topical therapy and phototherapy | 10 (8.2) | 2 (1.6) | 2 (3.1) | 0 (0.0) | 0 (0.0) | 0 (0.0) |
| ***Biologic drugs*** | **0 (0.0)** | **0 (0.0)** | **0 (0.0)** | **0 (0.0)** | **0 (0.0)** | **0 (0.0)** |
| ***Medical intervention*** |  |  |  |  |  |  |
| GMA | 0 (0.0) | 0 (0.0) | 0 (0.0) | 0 (0.0) | 0 (0.0) | 0 (0.0) |
| Tonsillectomy | 2 (0.2) | 2 (0.4) | 1 (0.3) | 0 (0.0) | 0 (0.0) | 0 (0.0) |
| **Severity: Severe to moderate** | **N=1228** | **N=949** | **N=722** | **N=531** | **N=363** | **N=246** |
| ***Topical therapy*** | **409 (33.3)** | **371 (39.1)** | **281 (38.9)** | **228 (42.9)** | **150 (41.3)** | **102 (41.5)** |
| ***Phototherapy*** | **37 (3.0)** | **43 (4.5)** | **30 (4.2)** | **18 (3.4)** | **8 (2.2)** | **10 (4.1)** |
| Without any additional prescription | 3 (8.1) | 10 (23.3) | 3 (10.0) | 5 (27.8) | 3 (37.5) | 4 (40.0) |
| With Topical therapy | 34 (91.9) | 33 (76.7) | 27 (90.0) | 13 (72.2) | 5 (62.5) | 6 (60.0) |
| ***Non-biologic drugs*** | **780 (63.5)** | **531 (56.0)** | **406 (56.2)** | **285 (53.7)** | **204 (56.2)** | **133 (54.1)** |
| Without any additional prescription | 105 (13.5) | 236 (44.4) | 224 (55.2) | 156 (54.7) | 124 (60.8) | 85 (63.9) |
| With phototherapy | 1 (0.1) | 2 (0.4) | 3 (0.7) | 2 (0.7) | 1 (0.5) | 1 (0.8) |
| With Topical therapy | 559 (71.7) | 255 (48.0) | 162 (39.9) | 119 (41.8) | 71 (34.8) | 41 (30.8) |
| With Topical therapy and phototherapy | 115 (14.7) | 38 (7.2) | 17 (4.2) | 8 (2.8) | 8 (3.9) | 6 (4.5) |
| ***Biologic drugs*** | **2 (0.2)** | **2 (0.2)** | **3 (0.4)** | 0 (0.0) | 0 (0.0) | **1 (0.4)** |
| Without any additional prescription | 0 (0.0) | 1 (50.0) | 0 (0.0) | 0 (0.0) | 0 (0.0) | 0 (0.0) |
| With non-biologic drugs | 1 (50.0) | 1 (50.0) | 1 (33.3) | 0 (0.0) | 0 (0.0) | 1 (100.0) |
| With Topical therapy | 0 (0.0) | 0 (0.0) | 1 (33.3) | 0 (0.0) | 0 (0.0) | 0 (0.0) |
| With phototherapy | 0 (0.0) | 0 (0.0) | 0 (0.0) | 0 (0.0) | 0 (0.0) | 0 (0.0) |
| With Topical therapy and phototherapy | 0 (0.0) | 0 (0.0) | 0 (0.0) | 0 (0.0) | 0 (0.0) | 0 (0.0) |
| With non-biologic drugs and phototherapy | 1 (50.0) | 0 (0.0) | 1 (33.3) | 0 (0.0) | 0 (0.0) | 0 (0.0) |
| With prescriptions of topical and non-biologics | 0 (0.0) | 0 (0.0) | 0 (0.0) | 0 (0.0) | 0 (0.0) | 0 (0.0) |
| With prescriptions of topical, non-biologics drugs and Phototherapy | 0 (0.0) | 0 (0.0) | 0 (0.0) | 0 (0.0) | 0 (0.0) | 0 (0.0) |
| ***Medical intervention*** |  |  |  |  |  |  |
| GMA | 0 (0.0) | 0 (0.0) | 0 (0.0) | 0 (0.0) | 0 (0.0) | 0 (0.0) |
| Tonsillectomy | 0 (0.0) | 2 (0.2) | 2 (0.3) | 0 (0.0) | 1 (0.3) | 0 (0.0) |
