# Supplementary material for: Treatment patterns and healthcare resource utilization in palmoplantar pustulosis patients in Japan: A claims database study
Source: PLoS One. 2020 May 22;15(5):e0232738. doi: 10.1371/journal.pone.0232738 (PMC7244105; doi:10.1371/journal.pone.0232738)
Supplement: S2 Table — (DOCX) [file pone.0232738.s002.docx]

**Table S2: List of drugs prescribed to treatment naïve PPP patients up to 6^th^ line of treatment**

| **Drug class** | **Drug name** | **ATC code** | **DDD** | **Units** | **Route of administration** |
| --- | --- | --- | --- | --- | --- |
| **Biologic drugs** | Adalimumab (Genetical Recombination) | L04AB04 | 2.9 | mg | Parenteral |
|  | Infliximab (Genetical Recombination) | L04AB02 | 3.75 | mg | Parenteral |
|  | Tocilizumab (Genetical Recombination) | L04AC07 | 20 | mg | Parenteral |
| **Non-biologic drugs** | Biotin | A11HA05 | - | - | - |
|  | Clarithromycin | J01FA09 | 0.5  1.0 | g | Oral  Parenteral |
|  | Minocycline | J01AA08 | 0.2 | g | Oral  Parenteral |
|  | Corticosteroids for systemic use, combinations | H02BX01 | - | - | - |
|  | Betamethasone | H02AB01 | 1.5  1.5  0.4 | mg | Oral  Parenteral  Parenteral (depot) |
|  | Prednisolone | D07AA03  D07XA02 | - | - | - |
|  |  | H02AB06 | 10  10 | mg | Oral  Parenteral |
|  | Dexamethasone | D07AB19  D07XB05  D10AA03 | - | - | - |
|  |  | H02AB02 | 1.5  1.5 | mg | Oral  Parenteral |
|  | Azithromycin | J01FA10 | 0.3  0.5 | g | Oral  Parenteral |
|  | Roxithromycin | J01FA06 | 0.3 | g | Oral |
|  | Methylprednisolone | D07AA01  D10AA02 | - | - | - |
|  |  | H02AB04 | 7.5  20 | mg | Oral  Parenteral |
|  | Triamcinolone | D07AB09  D07XB02 | - | - | - |
|  |  | H021B08 | 7.5  7.5 | mg | Oral  Parenteral |
|  | Hydrocortisone | D07AA02  D07XA01 | - | - | - |
|  |  | H02AB09 | 30  30 | mg | Oral  Parenteral |
|  | Etretinate | D05BB01 | 35 | mg | Oral |
|  | Doxycycline | J01AA02 | 0.1  0.1 | g | Oral  Parenteral |
|  | Alfacalcidol | A11CC03 | 1  1 | mcg | Oral  Parenteral |
|  | Ciclosporin | L04AD01 | 0.25  0.25 | g | Oral  Parenteral |
|  | Clindamycin | D10AF01 | - | - | - |
|  |  | J01FF01 | 1.2  1.8 | g | Oral  Parenteral |
|  | Colchicine | L01CC01 | - | - | - |
|  | Alendronic acid | M05BA04 | 10 | mg | Oral |
|  | Bisphosphonates | M05BA01 | 0.4  1.5 | g | Oral  Parenteral (course dose) |
|  |  | M05BA02 | 1.6  1.5 | g | Oral  Parenteral (course dose) |
|  |  | M05BA03 | 60 | mg | Parenteral (course dose) |
|  |  | M05BA04 | 10 | mg | Oral |
|  |  | M05BA05 | 0.4 | g | Oral |
|  |  | M05BA06 | 5  6 | mg | Parenteral (course dose) |
|  |  | M05BA07 | 5 | mg | Oral |
|  |  | M05BA08 | 4 | mg | Parenteral |
|  | Sulfasalazine | A07EC01 | 2  2 | g | Oral  Parenteral |
|  | Vitamin d and analogues | A11CC01 | - | - | - |
|  |  | A11CC02 | 1 | mg | Oral |
|  |  | A11CC03 | 1  1 | mcg | Oral  Parenteral |
|  |  | A11CC04 | 1  1 | mcg | Oral  Parenteral |
|  |  | A11CC05 | 20 | mcg | Oral |
|  |  | A11CC06 | - | - | - |
|  | Lincomycin | J01FF02 | 1.8  1.8 | g | Oral  Parenteral |
|  | Risedronic acid | M05BA07 | 5 | mg | Oral |
|  | Methotrexate | L01BA01 | - | - | - |
|  |  | L04AX03 | 2.5  2.5 | mg | Oral  Parenteral |
|  | Tetracycline | J01AA01 | 0.6 | g | Oral |
|  |  | J01AA02 | 0.1  0.1 | g | Oral  Parenteral |
|  |  | J01AA03 | 1 | g | Oral |
|  |  | J01AA04 | 0.6  0.6 | g | Oral  Parenteral |
|  |  | J01AA05 | 0.6 | g | Oral |
|  |  | J01AA06 | 1  1 | g | Oral  Parenteral |
|  |  | J01AA07 | 1  1 | g | Oral  Parenteral |
|  |  | J01AA08 | 0.2  0.2 | g | Oral  Parenteral |
|  |  | D06AA04 | - | - | - |
|  | Dapsone | D10AX05 | - | - | - |
|  |  | J04BA02 | 50 | mg | Oral |
|  | Erythromycin | J01FA01 | 1  2  1 | g | Oral  Oral  Parenteral |
|  | Bucillamine | M01CC02 | - | - | - |
|  | Calcitriol | A11CC04 | 1  1 | mcg | Oral  Parenteral |
|  |  | D05AX03 | - | - | - |
|  | Ibandronic acid | M05BA06 | 5  6 | mg | Oral  Parenteral (course dose) |
|  | Josamycin | J01FA07 | 2 | g | Oral |
|  | Other specific antirheumatic agents | M01CX | - | - | - |
|  | Vitamin k | B02BA01 | 20  20 | mg | Oral  Parenteral |
|  |  | B02BA02 | 10  2 | mg | Oral  Parenteral |
|  | Zoledronic acid | M05BA08 | 4 | mg | Parenteral |
| **Topical therapy** | Betamethasone | D07AC01 | - | - | - |
|  | Other antipsoriatics for topical use | D05AX | - | - | - |
|  | Clobetasol | D07AD01 | - | - | - |
|  | Betamethasone and antibiotics | D07CC01 | - | - | - |
|  | Difluprednate | D07AC19 | - | - | - |
|  | Diflorasone | D07AC10 |  |  |  |
|  | Dexamethasone | D07AB19  D07XB05  D10AA03 | - | - | - |
|  | Hydrocortisone butyrate | D07AB02 | - | - | - |
|  | Diflucortolone | D07AC06  D07XC04 |  |  |  |
|  | Tacalcitol | D05AX04 |  |  |  |
|  | Prednisolone | D07AA03  D07XA02 |  |  |  |
|  | Other dermatologicals | D11AX01  D11AX02  D11AX03  D11AX04  D11AX05  D11AX06  D11AX07  D11AX08 | -  0.4  -  -  -  -  -  - | -  g  -  -  -  -  -  - | -  Oral  -  -  -  -  -  - |
|  | Diphenhydramine | D04AA32 | - | - | - |
|  | Fludroxycortide | D07AC07 | - | - | - |
|  | Mometasone | D07AC13  D07XC03 | - | - | - |
|  | Clobetasone | D07AB01 | - | - | - |
|  | Fluocinonide | D07AC08 | - | - | - |
|  | Corticosteroids, potent (group iii) | D07AC01  D07AC02  D07AC03  D07AC04  D07AC05  D07AC06  D07AC07  D07AC08 | - | - | - |
|  | Calcipotriol, combinations | D05AX52 | - | - | - |
|  | Calcipotriol | D05AX02 | - | - | - |
|  | Alclometasone | D07AB10 | - | - | - |
|  | Hydrocortisone | D07AA02  D07XA01 | - | - | - |
|  | Hydrocortisone and antibiotics | D07CA01 | - | - | - |
|  | Beclometasone | D07AC15 | - | - | - |
|  | Other antibiotics for topical use | D06AX01  D06AX02  D06AX03  D06AX04  D06AX05  D06AX06  D06AX07  D06AX08 | - | - | - |
|  | Triamcinolone | D07AB09  D07XB02 | - | - | - |
|  | Amcinonide | D07AC11 | - | - | - |
|  | Fluocinolone acetonide | D07AC04 | - | - | - |
|  | Fluocinolone acetonide and antibiotics | D07CC02 | - | - | - |
|  | Prednisolone and antibiotics | D07CA03 | - | - | - |
|  | Triamcinolone and antibiotics | D07CB01 | - | - | - |
| g= gram  mg= milligram  mcg=microgram  ATC= Anatomic Therapeutic Chemical  DDD= Defined Daily Dose  - = no defined record | | | | | |
