# Supplementary material for: Treatment patterns and healthcare resource utilization in palmoplantar pustulosis patients in Japan: A claims database study
Source: PLoS One. 2020 May 22;15(5):e0232738. doi: 10.1371/journal.pone.0232738 (PMC7244105; doi:10.1371/journal.pone.0232738)
Supplement: S3 Table — (DOCX) [file pone.0232738.s003.docx]

**Table S3: List of drugs approved for the treatment of PPP in Japan**

| **Drug name** | **ATC code** |
| --- | --- |
| Dexamethasone | D00292 |
| Dexamethasone phosphate sodium | D00975 |
| Betamethasone sodium phosphate ester | D00972 |
| Triamcinolone acetonide | D00983 |
| Cyclosporine | D00184 |
| Beclomethasone propionate | D00689 |
| Mometasone furoate | D00690 |
| Hydrocortisone | D00088 |
| Betamethasone | D00244 |
| Prednisolone | D00472 |
| Dexamethasone propionate | D01632 |
| Dexamethasone valerate | D01948 |
| Hydrocortisone butyrate | D01619 |
| Hydrocortisone butyrate propionate | D01886 |
| Prednisolone valerate acetate | D03301 |
| Betamethasone valerate | D01357 |
| Betamethasone dipropionate | D01637 |
| Betamethasone butyrate propionate | D02032 |
| Fluocinolone acetonide | D01825 |
| Fluocinonide | D00325 |
| Alclomethasone propionate | D01820 |
| Diflucortron valerate | D01764 |
| Fludroxycortide | D00328 |
| Diflorazone acetate | D01327 |
| Amcinonide | D01387 |
| Difluprednate | D01266 |
| Clobetasol propionate | D01272 |
| Deprodone propionate | D01434 |
| Betamethasone valerate ester / gentamicin sulfate | D04773 |
| Fradiomycin sulfate / fluocinolone acetonide | D04795 |
| Hydrocortisone acetate / Diphenhydramine hydrochloride / Fradiomycin sulfate | D04806 |
| Defatted soybean carbonized tar | D08734 |
| Salicylic acid | D00097 |
| Tacalcitol hydrate | D01472 |
| Maxacalcitol | D01098 |
| Etretinate | D00316 |
| Guselkumab | D10438 |
