# Supplementary material for: Treatment patterns and healthcare resource utilization in palmoplantar pustulosis patients in Japan: A claims database study
Source: PLoS One. 2020 May 22;15(5):e0232738. doi: 10.1371/journal.pone.0232738 (PMC7244105; doi:10.1371/journal.pone.0232738)
Supplement: S4 Table — (DOCX) [file pone.0232738.s004.docx]

**Table S4: Overall duration of treatment lines, number of treatment-related events up to 6^rd^ line of treatment according to the treatment regimen dispensed, (N) total number of patients in the cohort; data are reported as N (%) percentages.**

|  | **Topical therapy** | **Phototherapy** | **Non-biologic drugs** | **Biologic drugs ^a^** |
| --- | --- | --- | --- | --- |
| **All treatment lines** | **3258** | **288** | **2966** | **9** |
| Duration of the line |  |  |  |  |
| Mean (SD) | 77.73 (116.2) | 119.03 (150.7) | 101.5 (231.8) | 156.0 (104.1) |
| Median | 28 | 60 | 28 | 162 |
| Treatment-related events |  |  |  |  |
| > Discontinuation | 620 (19.0%) | 38 (13.2%) | 587 (19.8%) | 1 (11.1%) |
| > Switch | 2305 (70.8%) | 208 (72.2%) | 1945 (65.6%) | 6 (66.7%) |
| > Add-on | 0 | 0 | 0 | 0 |
| > Reduction | 0 | 0 | 0 | 0 |
| > No change | 333 (10.2%) | 42 (14.6%) | 434 (14.6%) | 2 (22.2%) |
| *All medical interventions were excluded*.  *^a^ Biologics were not use for PPP condition at this investigation time frame.* | | | | |
